# Supplementary material for: Influence of frailty on cardiovascular events and mortality in patients with Chronic Obstructive Pulmonary Disease (COPD): Study protocol for a multicentre European observational study
Source: PLoS One. 2024 Jun 25;19(6):e0300945. doi: 10.1371/journal.pone.0300945 (PMC11198743; doi:10.1371/journal.pone.0300945)
Supplement: S1 File — (ZIP) [file pone.0300945.s001.zip › Supporting Information File S1/COPD frailty mortality CV protocol v1 28.03.23.docx]

**Frailty and its influence on cardiovascular events and mortality in patients with Chronic Obstructive Pulmonary Disease (COPD): A multicentre observational study.**

Verduri A^1^, Clini E^1^, Hewitt J^2^

Department of Surgical and Medical Sciences, Respiratory Unit, Policlinico Modena, University of Modena and Reggio Emilia, Modena (Italy) ^1^

Division of Population Medicine, Cardiff University (UK) ^2^

**Chief Investigator**: Alessia Verduri, Department of Surgical and Medical Sciences, Respiratory Unit, Policlinico Modena, University of Modena and Reggio Emilia, Modena (Italy)

# KEY STUDY CONTACTS

| Chief Investigator | Alessia Verduri |
| --- | --- |
| Sponsor | n/a |
| Funder(s) | n/a |
| Key Protocol Contributors | Alessia Verduri, Enrico Clini, Jonathan Hewitt |
| Trial Committee | Alessia Verduri, Enrico Clini, Jonathan Hewitt |

**STUDY SUMMARY**

| Study Title | Influence of frailty on cardiovascular events and mortality in patients with COPD |
| --- | --- |
| Study Design | Observational |
| Study Participants | Stable COPD outpatients |
| Planned Size of Sample (if applicable) | 300 |
| Follow up duration (if applicable) | 24 months |
| Planned Study Period | 24 months |
| Research Question/Aim(s) | To assess frailty and number of cardiovascular events in adult patients with COPD in stable condition. To correlate frailty with long-term cardiovascular mortality, all-cause mortality, and mortality due to COPD in COPD patients.  A further research question is the prevalence of frailty in COPD. |

**ROLE OF STUDY SPONSOR AND FUNDER**

This study project is unfunded.

**ROLES AND RESPONSIBILITIES OF STUDY MANAGEMENT COMMITEES/GROUPS & INDIVIDUALS**

Trial Steering Committee (TSC):

This will be led by Alessia Verduri, Enrico Clini, Jonathan Hewitt and the research team. These are observational data and there are minimal safety issues expected.

Patient and Public Involvement (PPI) Group

It will be possible to develop a PPI group of patients who have participated to the study. Towards the end of the study, the coordinator centre will invite a group of patients back to the hospital to discuss their personal opinion about the meaning of the research study.

**Study Protocol**

**Background**

Frailty is a clinical syndrome that increases vulnerability to stressors due to decline of multiple physiological reserves. People living with frailty area at higher risk of falls, disability, long hospitalisations, access to care homes, and death. [1] There are two principal approaches of identification of frailty: the deficit accumulation model and the phenotype model. [1,2] Based on these models, different tools have been created and validated to assess frailty in clinical practice. [3]

Chronic diseases such as Chronic Obstructive Pulmonary Disease (COPD) increase the risk of frailty. [3,4] Literature on frailty in COPD have been emerging in the recent years. While frailty is not synonym of ageing, it is well known that people over 65 years are more likely to live with frailty. [4] In addition, prevalence of COPD increases with age. [5] A double probability of being frail has been demonstrated in patients with COPD compared to patients without COPD in the paper of Marengoni et al. published in 2018. The study reported a prevalence of frailty in COPD between 9% and 64% based on the phenotype model and from 9% to 28% using different frailty assessment instruments. [6] Recent research studies have shown that frailty can increase the risk of mortality in patients with confirmed diagnosis of COPD according to GOLD guidelines ([www.goldcopd.org](http://www.goldcopd.org)). [7-11]

Additionally, the risk of cardiovascular events is higher after COPD exacerbation. Cardiovascular comorbidities are very common in patients with COPD and can increase the risk of death in this population. [5,12]

The present protocol describes a research study project on patients with COPD to investigate the influence that frailty can have on cardiovascular risk in these patients. The project will assess the association between frailty and all-cause mortality and the relationship between frailty and cardiovascular mortality. Our team have widely collected data on frailty previously using routinely collected service evaluation level data.

The study has the goal to provide relevant data for clinical practice and management of patients with COPD to improve quality of care, and potentially prognosis, considering a more accurate characterisation of the single patient severity.

**Aims**

To assess whether frailty correlates with clinical outcomes in patients diagnosed with COPD according to guidelines.

**Methods**

Study design: a multicentre observational study.

Study setting: two hospitals in Italy, other than the principal investigator site of Modena, that provide care for outpatients diagnosed with COPD have been invited to participate. The central study team subsequently provided the ethical approval, protocol, central organisation and long-term delivery of the project.

Participants: The study participants will be outpatients with COPD attending the selected hospitals for scheduled visit. The diagnosis of COPD will be according to the GOLD guidelines ([www.goldcopd.org](http://www.goldcopd.org)) and confirmed by a post-bronchodilator FEV_1_/FVC < 0.70.

Inclusion criteria:

- Patients with age ≥18 years, with diagnosis of COPD of at least 1 year, in stable condition, without acute exacerbation in the last 30 days and/or all-cause hospitalisation in the last 3 months
- Smoking history of ≥ 10 pack/years
- Diagnosis of COPD according to GOLD guidelines ([www.goldcopd.org](http://www.goldcopd.org))
- Individuals able to provide their written informed consent.

Exclusion criteria:

- Concurrent diagnosis of asthma
- Concurrent diagnosis of interstitial lung disease
- Presence of restrictive pattern on spirometry
- No history of smoking habit
- Individuals not able to provide their written informed consent.

**Principal investigators**

The principal investigators at each participating site are responsible for organising and leading the local research teams. They will submit relevant documents to local Ethics Committee for approval and ensured that collaborators act in accordance with local clinical governance and guidelines.

**Patient identification and data collection**

Outpatients will be screened for inclusion criteria by the local team. Each site Principal Investigator (PI) will have Good Clinical Practice (GCP) training and will have local responsibility for data quality and entry. To ensure data quality each PI and member of the clinical team will be required to watch a short training video on completion of the Clinical Frailty Scale 1-9.

Data collection will be carried out using the case report form (CRF) presented in the supplementary appendix. Where personal information is collected at each site, it will be kept secure, and maintained. This will involve the creation of a number code for each patient (Patient Identification, PID) that will guarantee data anonymisation.

**Baseline data**

- Age, sex, occupation (current or previous), body mass index (BMI, Kg/M^2^), active smoking expressed as pack/years (= number of cigarettes per day multiplied by the number of years the person has smoked/20), years of COPD, GOLD classes (1, 2, 3, 4) and GOLD grades based on GOLD 2011 (A, B, C, D) and GOLD 2023 reports (A, B, E) ([www.goldcopd.org](http://www.goldcopd.org)), frailty score using the Clinical Frailty Scale (**Figure 1**), list and number of comorbidities, list and number of drugs taken.

**Clinical data at follow up (12 and 24 months)**

- Number of cardiovascular events.
- Number of all-cause deaths.
- Number of deaths due to cardiovascular cause.
- Number of deaths due to COPD.
- Frailty score using the Clinical Frailty Scale (**Figure 1**).
- Follow up spirometry.

**Frailty assessment**

We will use the Clinical Frailty Score. This has been validated for use to assess frailty in older patients. [13] The score ranks from 1 to 9 with a score of ≥5 being classed as frail and 9 as terminally ill.


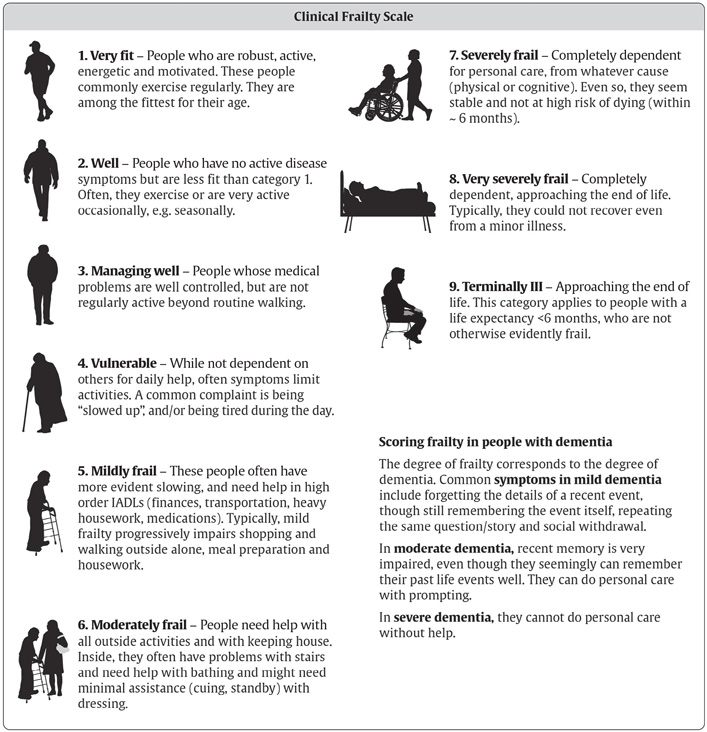


**Figure 1. Clinical Frailty Scale.**

**Primary outcome**

- Number of cardiovascular events (including hospital admissions for cardiovascular events) at 12 and 24 months in COPD patients living with frailty *vs* non-frail COPD patients.

**Secondary outcomes**

- All-cause long-term mortality at 12 and 24 months in COPD patients living with frailty *vs* non-frail COPD patients;
- Long-term cardiovascular mortality at 12 and 24 months in COPD patients living with frailty *vs* non-frail COPD patients;
- Prevalence of frailty in COPD
- Number of deaths due to COPD.

**Quality assurance**

The study will be registered (www.clinicaltrials.gov). The quality of this study has been assessed by peer review by professionals with relevant expertise (respiratory physicians, geriatricians, statisticians).

**Safety reporting/adverse events**

These are routinely collected clinical data, we do not predict any adverse events.

**Validation Data**

Validation will be performed by local teams on 25% of data fields for 10% of cases. The validated fields will include key demographic and outcome data.

**Data management, Data protection and patient confidentiality**

All investigators and trial site staff will comply with the requirements of the Good Clinical Practice (GCP) and Guidelines for Data Processing (GDPR 679/2016) with regards to the collection, storage, processing and disclosure of personal information.

Where personal information is collected, it will be kept secure, and maintained. This will involve:

- The creation of coded, depersonalised data where the participant’s identifying information is replaced by an unrelated sequence of characters
- Secure maintenance of the data and the linking code in separate locations using encrypted digital files within password protected folders and storage media
- Limiting access to the minimum number of individuals necessary for quality control, audit, and analysis
- Confidentiality of data will be preserved when the data are transmitted to sponsors and co-investigators as patients will be assigned a study ID number
- Data collected from sites will be via the electronic database
- Data custodian is Dr Alessia Verduri
- Only researchers who are directly involved in the data analysis will have full access to the study dataset.

**Access to the final study dataset**

The research team will have full access to the study dataset in order to ensure that the overall results are not disclosed by an individual trial site prior to the main publication.

The following individuals involved in the trial will have access to the full dataset: Dr Alessia Verduri, Professor Enrico Clini.

Possibilities for secondary analysis will be included into the initial participant consent. Sites may request access to the full dataset from the Chief Investigator.

The research team are experienced in data management. To that end, the simplest form of data collection is via secure transfer of anonymised data to a central study team, using freely available software such as Microsoft XL. Password-protected login details will be provided to local collaborators permitting secure data entry into a centrally held database. This database with be held within University of Modena (Italy) and stored for a minimum of 7 years.

No patient identifiable information will be uploaded or stored on the secure database. Collaborators will anonymise patients by recording patient clinical numbers alongside database numbers in a separate secure spreadsheet to aid the collection of data locally.

**Statistical analysis and power calculation**

We will aim to recruit a minimum of 300 patients; 100 patients across a minimum of 3 sites.

Due to the importance of data on mortality in COPD population it is envisaged that interim analyses will be conducted as data are accumulated.

This protocol and analysis will be drafted by a medical statistician fully blinded to the outcome data.

Data will be analysed for correlation between frailty and clinical outcomes. The primary analysis will be the number of cardiovascular events (time to event) at 12 and 24 months, adjusting for sex, age (18-64, 65-80, >80) and frailty at the time of recruitment, and other clinically relevant covariates.

The secondary analyses will assess the time to all-cause mortality, cardiovascular mortality, and mortality due to COPD that will be fitted to compare those classified as frail *vs* not frail visually using a Kaplan Meier plot. Data will be analysed using a multivariable Cox baseline proportional hazards regression, adjusting for covariates. The time to mortality will be calculated from the date of study entry to the end of follow up. The analyses will be presented as Odds Ratio (adjusted OR) with associated 95% confidence intervals and p-values. Subgroups analyses (age, sex, GOLD classes and grades, comorbidities) will be conducted to assess the grade of frailty.

**Ethics**

The approval from the Ethics Committee of the Coordinator Centre has not yet been obtained. The evaluation of the research protocol is expected by the 5^th^ September 2023.

**Registration**

The data that are being recorded are routine data. Following proportional ethical approval from the Ethics Committee of University of Modena (Italy), it will be approved and opened at each participating site. All participating units must obtain approval from their local Ethics Committee.

**Dissemination**

All data will be reported as a whole cohort. Unit level data for comparison will be fed back to collaborators to support local service improvement. This project will be submitted for presentation at a national or international Respiratory and Geriatric conference. Manuscript(s) will be prepared following close of the project.

**References**

1. Fried LP, Tangen CM, Walston J, *et al.* Frailty in older adults: evidence for a phenotype. *J Gerontol A Biol Sci Med Sci* 2001*;* 56(3): M146-56.
2. Mitnitski AB, Mogilner AJ, Rockwood K. Accumulation of deficits as a proxy measure of aging. *ScientificWorldJournal* 2001; 1:323-36.
3. Clegg A, Young J, Iliffe S, *et al*. Frailty in elderly people. *Lancet* 2013; 381 (9868):752-62.
4. Morley JE, Vellas B, van Kan GA, *et al.* Frailty consensus: a call to action. *J Am Med Dir Assoc* 2013; 14(6): 392-7.
5. Global Initiative for Chronic Obstructive Lung Disease (www.goldcopd.org).
6. Marengoni A Vetrano DL, Manes-Gravina E, *et al*. The Relationship Between COPD and Frailty: A Systematic Review and Meta-Analysis of Observational Studies. *Chest* 2018; 154(1):21-40.
7. Luo J, Zhang D, Tang W, *et al*. Impact of Frailty on the Risk of Exacerbations and All-Cause Mortality in Elderly Patients with Stable Chronic Obstructive Pulmonary Disease. *Clin Interv Aging* 2021; 16:593-601.
8. Kennedy CC, Novotny PJ, LeBrasseur NK, *et al*. Frailty and Clinical Outcomes in Chronic Obstructive Pulmonary Disease. *Ann Am Thorac Soc* 2019; 16(2):217-224.
9. Lee SY, Nyunt MSZ, Gao Q, *et al*. Co-occurrence of Physical Frailty and COPD and Association with Disability and Mortality: Singapore Longitudinal Ageing Study. *Chest* 2022; 161(5):1225-1238.protoco
10. Galizia G, Cacciatore F, Testa G, *et al*. Role of clinical frailty on long-term mortality of elderly subjects with and without chronic obstructive pulmonary disease. Aging Clin Exp Res 2011; 23:118-125.
11. Lahousse L, Ziere G, Verlinden VJA, *et al*. Risk of frailty in elderly with COPD: a population-based study. J Gerontol A Biol Sci Med Sci 2016; 71(5):689-695.
12. Divo M, Cote C, de Torres JP, *et al*. Comorbidities and risk of mortality in patients with chronic obstructive pulmonary disease. Am J Respir Crit Care Med 2012; 186:155-161.
13. Rockwood K, Song X, MacKnight C, *et al.* A global clinical measure of fitness and frailty in elderly people. *CMAJ* 2005; 173(5):489-95.
