# Supplementary material for: Influence of frailty on cardiovascular events and mortality in patients with Chronic Obstructive Pulmonary Disease (COPD): Study protocol for a multicentre European observational study
Source: PLoS One. 2024 Jun 25;19(6):e0300945. doi: 10.1371/journal.pone.0300945 (PMC11198743; doi:10.1371/journal.pone.0300945)
Supplement: S1 File — (ZIP) [file pone.0300945.s001.zip › Supporting Information File S1/Informed Consent Form.doc]

# Informed Consent Form

**Study: Influence of frailty on cardiovascular events and mortality in patients with Chronic Obstructive Pulmonary Disease (COPD): Study Protocol for a multicentre European observational study.**

Participant ID:

We are doing research on COPD, which is very common in this country. I am going to give you information and invite you to be part of this research study. You do not have to decide today whether or not you will participate in the research. Before you decide, you can talk to anyone you feel comfortable with about the research. There may be some words that you do not understand. Please ask me to stop as we go through the information and I will take time to explain. If you have questions later, you can ask them of me, or the staff.

COPD has been associated with the presence of frailty and the risk of cardiovascular disease. Frailty is a clinical condition that may increase your vulnerability to acute events. People living with frailty can be at higher risk of falls and disability. They can have prolonged hospitalization for any cause and high risk of fatal events. We are doing this research study to investigate the influence that frailty may have on your risk of cardiovascular events and whether the presence of frailty can be associated with admissions or fatal events for cardiovascular problems.

This research study will involve only a spirometry, no drugs will be tested, and no blood samples will be taken. We are inviting all adults with COPD who attend the Respiratory Outpatient Department to participate in the study.

Whether you choose to participate or not, all the services you receive at this clinic will continue and nothing will change. If you choose not to participate in this research study, you will offered the treatment that is routinely offered in this hospital for COPD. You may also change your mind later and stop participating even if you agreed earlier.

During the research you make three visits to the clinic, the second will be after one year, and the third after two years. In total, you will be asked to come three times to the clinic in two years. At the end of two years, the research study will be finished.

There may not be any benefit for you but your participation is likely to help us find the answer to the research question. You will not be given any other money or gifts to take part in this research study.

The information that we collect from this research study will be kept confidential. Information about you that will be collected during the study will be put away and no-one but the researchers will be able to see it. Any information about you will have a number on it instead of your name. Only the researchers will know what your number is. There will be meetings in the community and these will be announced. After these meetings, we will publish the results in order that other interested people may learn from our research.

I have read the foregoing information, or it has been read to me. I have had the opportunity to ask questions about it and any questions that I have asked have been answered to my satisfaction. I consent voluntarily to participate as a participant in this research study.

**Print Name of Participant__________________**

**Signature of Participant ___________________**

**Date ___________________________**

**Day/Month/Year**

**If illiterate**

I have witnessed the accurate reading of the consent form to the potential participant, and the individual has had the opportunity to ask questions. I confirm that the individual has given consent freely.

**Print name of witness_____________________**

**Signature of witness ______________________**

**Date ________________________**

**Day/Month/Year**

Statement by the person taking consent

I have accurately read out the information to the potential participant, and to the best of my ability made sure that the participant understands that the following assessments will be done:

1. Spirometry and bronchodilation test

2. Clinical Frailty Scale

3. Health data collection.

I confirm that the participant was given an opportunity to ask questions about the study, and all the questions asked by the participant have been answered correctly and to the best of my ability. I confirm that the individual has not been coerced into giving consent, and the consent has been given freely and voluntarily.

**A copy of this Informed Consent Form has been provided to the participant.**

**Print Name of** **/person taking the consent________________________**

**Signature of person taking the consent__________________________**

**Date ___________________________**

**Day/Month/Year**
