# Supplementary material for: Influence of frailty on cardiovascular events and mortality in patients with Chronic Obstructive Pulmonary Disease (COPD): Study protocol for a multicentre European observational study
Source: PLoS One. 2024 Jun 25;19(6):e0300945. doi: 10.1371/journal.pone.0300945 (PMC11198743; doi:10.1371/journal.pone.0300945)
Supplement: S1 File — (ZIP) [file pone.0300945.s001.zip › Supporting Information File S1/SPIRIT-Checklist.doc]

SPIRIT 2013 Checklist: Recommended items to address in a clinical trial protocol and related documents*

| Section/item | ItemNo | Description |
| --- | --- | --- |
| **Administrative information** | | |
| Title | 1 | Influence of frailty on cardiovascular events and mortality in patients with Chronic Obstructive Pulmonary Disease (COPD): Study Protocol for a multicentre European observational study. |
| Trial registration | 2a | NCT05922202 ([www.clinicaltrials.gov](http://www.clinicaltrials.gov/)). |
|  | 2b | University of Modena and Reggio Emilia Ethics Committee Area Vasta Nord Emilia-Romagna (Modena, Italy); approval number 448/2023/OSS/AOUMO – SIRER ID 6390. |
| Protocol version | 3 | Version 1.0, Date 28.03.23 |
| Funding | 4 | The study project is unfunded. |
| Roles and responsibilities | 5a | Alessia Verduri, Respiratory Unit, Department of Surgical and Medical Sciences, Policlinico Modena, University of Modena and Reggio Emilia, Modena (Italy); conceptualisation, investigation (Chief Investigator), methodology.  Enrico Clini, Respiratory Unit, Department of Surgical and Medical Sciences, Policlinico Modena, University of Modena and Reggio Emilia, Modena (Italy); investigation, methodology.  Ben Carter, Department of Biostatistics and Health Informatics, Institute of Psychiatry, Psychology and Neuroscience, King’s College London (UK); statistical considerations, methodology.  Jonathan Hewitt, Division of Population Medicine, Cardiff University (UK); investigation, methodology. |
|  | 5b | NA |
|  | 5c | NA |
|  | 5d | Only researchers who are directly involved in the data analysis will have full access to the study dataset. |
| Introduction |  |  |
| Background and rationale | 6a | Cardiovascular comorbidities are very common in patients with COPD and can increase the risk of death in this population. Reason for trial: lack of available evidence on the role of frailty in cardiovascular morbidity and mortality in COPD.  Research questions:   1) Number of cardiovascular events at 12 and 24 months in COPD patients living with frailty *vs* non-frail COPD patients. 2) Number of deaths due to cardiovascular cause at 12 and 24 months in COPD patients living with frailty vs non-frail COPD patients. 3) Number of deaths due to all-cause at 12 and 24 months in COPD patients living with frailty *vs* non-frail COPD patients. 4) Number of deaths due to COPD at 12 and 24 months in COPD patients living with frailty *vs* non-frail COPD patients. 5) Prevalence of frailty in COPD. |
|  | 6b | Exploring cardiovascular morbidity and mortality in non-frail COPD patients. |
| Objectives | 7 | To provide relevant data for management of patients with COPD, assessing whether frailty correlates with clinical outcomes. |
| Trial design | 8 | Multicentre European observational study, following the STROBE statement for reporting of cohort studies (https://www.strobe-statement.org). Exploratory framework. |
| Methods: Participants, interventions, and outcomes | | |
| Study setting | 9 | Two academic hospitals and one local health care service in Italy and hospital sites in the UK. Reference to where list of study sites can be obtained. |
| Eligibility criteria | 10 | Inclusion criteria for participants   1. Patients with age ≥18 years, with diagnosis of COPD of at least 1 year, in stable condition, without acute exacerbation in the last 30 days and/or all-cause hospitalisation in the last 3 months 2. Smoking history of ≥ 10 pack/years 3. Diagnosis of COPD according to GOLD guidelines ([www.goldcopd.org](http://www.goldcopd.org/)) 4. Individuals able to provide their written informed consent.   Exclusion criteria for participants   1. Concurrent diagnosis of asthma 2. Concurrent diagnosis of interstitial lung disease 3. Presence of restrictive pattern on spirometry 4. No history of smoking habit 5. Individuals not able to provide their written informed consent. |
| Interventions | 11a | NA |
|  | 11b | NA |
|  | 11c | NA |
|  | 11d | NA |
| Outcomes | 12 | Primary outcome   - Number of cardiovascular events (including hospital admissions for cardiovascular events) at 12 and 24 months in COPD patients living with frailty *vs* non-frail COPD patients.   Secondary outcomes   - Long-term cardiovascular mortality at 12 and 24 months in COPD patients living with frailty *vs* non-frail COPD patients. - All-cause long-term mortality at 12 and 24 months in COPD patients living with frailty *vs* non-frail COPD patients. - Mortality due to COPD at 12 and 24 months in COPD patients living with frailty *vs* non-frail COPD patients. - Prevalence of frailty in COPD.   Baseline variables  Age, sex, occupation (current or previous), body mass index (BMI, Kg/M^2^), active smoking expressed as pack/years (= number of cigarettes per day multiplied by the number of years the person has smoked/20), years of COPD, GOLD classes (1, 2, 3, 4) and GOLD grades based on GOLD 2011 (A, B, C, D) and GOLD 2023 reports (A, B, E) ([www.goldcopd.org](http://www.goldcopd.org/)), frailty score using the Clinical Frailty Scale (CFS), list and number of comorbidities, list and number of drugs taken.  The Clinical Frailty Scale has been validated for use to assess frailty in older patients. |
| Participant timeline | 13 | The **Figure 1** (SPIRIT schedule of enrolment, interventions, and assessments) has been included in the protocol. |
| Sample size | 14 | Estimated number of participants: minimum 320 subjects. Assuming the proportion of those that are not frail (40%), mildly frail (60%) and moderately to severely frail (75%) will exhibit at least one cardiovascular event at 1 year. In order to detect these two comparisons (not frail, versus mildly frail and not frail versus moderately frail), using a chi-squared test with type-1 error=0.025 and 80% power. We will need a minimum of 320 patients, approximately 120:120:80 for CFS 1-3, 4-5, 6-9 respectively. |
| Recruitment | 15 | Patients will be screened for inclusion criteria in outpatient settings by the local team. |
| **Methods: Assignment of interventions (for controlled trials)** | | |
| Allocation: |  |  |
| Sequence generation | 16a | NA |
| Allocation concealment mechanism | 16b | NA |
| Implementation | 16c | NA |
| Blinding (masking) | 17a | NA |
|  | 17b | NA |
| **Methods: Data collection, management, and analysis** | | |
| Data collection methods | 18a | Data collection will be carried out using the electronic case report form, presented in the Supporting Information file S1. No patient identifiable information will be uploaded or stored on the secure database. Spirometry will be performed according to the standard procedures. The researchers involved in the study have widely collected data on frailty previously using the CFS. |
|  | 18b | It will be possible to develop a Patient and Public Involvement group of patients who have participated to the baseline assessment for promoting participant complete follow-up. |
| Data management | 19 | The details of data management procedures can be found in the protocol. |
| Statistical methods | 20a | Statistical methods for analysing primary and secondary outcomes can be found in the protocol. |
|  | 20b | Subgroup analyses are explained in the protocol. |
|  | 20c | NA |
| **Methods: Monitoring** | | |
| Data monitoring | 21a | The data monitoring committee (DMC) will be led by Dr. Alessia Verduri, working closely with the investigators involved. These are observational data and there are minimal safety issues expected. |
|  | 21b | Due to the importance of data on mortality in COPD population it is envisaged that interim analyses will be conducted as data are accumulated. |
| Harms | 22 | As this study routinely collected clinical data, there are minimal safety issues expected. |
| Auditing | 23 | NA |
| Ethics and dissemination | | |
| Research ethics approval | 24 | The study was approved by the University of Modena and Reggio Emilia Ethics Committee Area Vasta Nord Emilia-Romagna (Modena, Italy); approval number 448/2023/OSS/AOUMO – SIRER ID 6390, 14^th^ November 2023. All participating units must obtain approval from their local Ethics Committee. In the UK, this study was deemed to be a service evaluation, individually approved for use in the UK hospital site. |
| Protocol amendments | 25 | No currently plans for protocol modifications are expected. |
| Consent or assent | 26a | The researchers at each site will obtain informed consent from potential participants. |
|  | 26b | NA |
| Confidentiality | 27 | Personal data will be kept secure and maintained, based on:   - The creation of coded, depersonalised data where the participant’s identifying information is replaced by an unrelated sequence of characters. - No patient identifiable information will be uploaded or stored on the secure database. Collaborators will anonymise patients by recording patient clinical numbers alongside database numbers in a separate secure spreadsheet to aid the collection of data locally. - Limiting access to the minimum number of individuals necessary for quality control and analysis. |
| Declaration of interests | 28 | None declared. |
| Access to data | 29 | Only researchers who are directly involved in the data analysis will have access to the study dataset. Participating sites may request access to the full final dataset from the Chief Investigator. |
| Ancillary and post-trial care | 30 | NA |
| Dissemination policy | 31a | This project will be submitted for presentation at a national or international Respiratory and Geriatric conference. Manuscript(s) will be prepared following close of the project. Manuscript(s) will also be prepared following interim data analysis at 12 months if numbers of patients recruited to the study at that time reach the planned sample size. |
|  | 31b | NA |
|  | 31c | No currently plans for public access to the full protocol, participant-level dataset, and statistical code. |
| Appendices |  |  |
| Informed consent materials | 32 | Patient consent form included in the Supporting Information file S1. |
| Biological specimens | 33 | NA |

*It is strongly recommended that this checklist be read in conjunction with the SPIRIT 2013 Explanation & Elaboration for important clarification on the items. Amendments to the protocol should be tracked and dated. The SPIRIT checklist is copyrighted by the SPIRIT Group under the Creative Commons “[Attribution-NonCommercial-NoDerivs 3.0 Unported](http://www.creativecommons.org/licenses/by-nc-nd/3.0/)” license.
